# Advances in Computational Drug Repurposing, Driver Genes, and Therapeutics in Lung Adenocarcinoma

**DOI:** 10.3390/biom15101373

**Published:** 2025-09-27

**Authors:** Sajjad Nematzadeh, Arzu Karaul

**Affiliations:** Software Engineering, Engineering and Natural Sciences, Istanbul Topkapi University, Istanbul 34087, Türkiye

**Keywords:** drug repurposing, computational drug repositioning, lung adenocarcinoma, driver genes

## Abstract

This review catalogs candidate LUAD driver genes and their roles, recent discoveries, and therapeutic avenues. Beyond experimental repurposing, we evaluate modern computational methods and how they complement bench work. We conclude by appraising recent LUAD repurposing studies through a computational lens, emphasizing practical integration into translational research. Highlights: Overview of drug repurposing methods: We provide a list of six experimental and a brief taxonomy of eight computational drug repurposing method families. Recent insights into LUAD driver genes: We present a curated panel of LUAD drivers mapped to pathways, with alteration types, functions, and therapeutic implications. LUAD-focused computational repurposing studies: We provide a synthesis of recent LUAD studies presenting clear method families, highlighting exemplar pipelines, prioritized candidate drugs, and datasets.

## 1. Drug Repurposing Review

Drug repurposing is the systematic discovery and validation of new clinical uses of different targets for approved or clinically tested drugs. It utilizes computational inference, experimental screening, real-world evidence, and human genetics. The most important applications repeatedly highlighted are oncology, immune and inflammatory diseases, neurologic and psychiatric disorders, cardiovascular and metabolic conditions, and rapid response to infectious outbreaks and rare diseases. Advantages emphasized across studies include shorter timelines and lower costs than de novo discovery and reuse of existing safety and manufacturing packages. In addition, a higher translational likelihood is achieved when supported by human data. Also, the ability to exploit polypharmacology for complex and multifactorial diseases is enhanced. Moreover, this approach extends the domain of transparent mechanistic hypotheses that guide trial design and patient stratification. Table 1 presents methods that are utilized in vitro, in the real world, and in experimental conditions.

In vitro repurposing tests involve directly applying approved or known compounds to cells or organoids to uncover phenotypes, clues about the mechanism of action, dose–response relationships, and basic toxicity. These methods are experimentally grounded but slower, more expensive, and sensitive to assay design, cell context, and the absence of full-body pharmacology. In contrast, in silico repurposing explores large chemical and biological spaces using omics signatures, structures, networks, and machine learning to prioritize candidates efficiently and at low cost. Additionally, it is fast and hypothesis-generating but relies on data quality and can be opaque or biased. Practically, in vitro provides causal, mechanistic evidence at the lab bench, while in silico offers a broad scope and hypothesis triage. As a result, the most effective workflow combines in silico approaches to rank and cluster candidates, with subsequent in vitro studies to confirm biology, refine doses, and select hits for further in vivo or clinical testing. Table 2 lists computational (in silico) drug repurposing methods.

**Figure 1:**
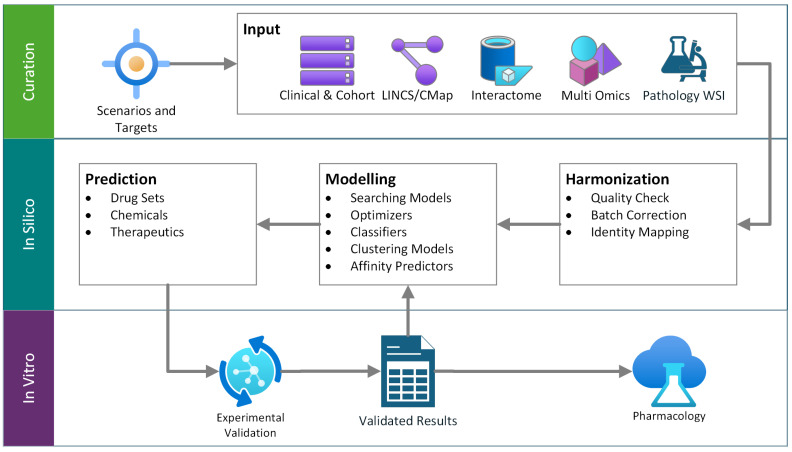
General workflow of the hybrid drug repurposing pipeline.

Figure 1 illustrates a schematic pipeline that uses both in silico and in vitro advantages. The pipeline focuses on a scenario-defined target and proposes a set of candidate drugs or therapeutics. Diverse data are curated and harmonized, then used in the model design phase. Results are tested and, when possible, validated in vitro; this cycle repeats until the desired accuracy is achieved. Final candidates proceed to drug design and pharmacological evaluation.

## 2. Cancer, Lung Adenocarcinoma, and Recent Therapeutics

Cancer is a disease in which transformed cells proliferate without normal control and evolve under natural selection, enabling invasion and metastasis [16]. Lung adenocarcinoma (LUAD) is the most common subtype of non-small-cell lung cancer (NSCLC) and usually begins in the distal lung epithelium of the alveoli, arising from alveolar epithelial cells (type II and, in some contexts, type I) and early transitional epithelial states [17,18,19]. Lung cancer remains the leading cause of cancer death worldwide. This disorder is a major cause of cancer mortality in the United States [20,21]. The disease mainly affects older adults, with median ages at diagnosis around the early 70s in high-income settings, though there is international variation by country and smoking prevalence [22]. Sex patterns are shifting: according to U.S. data published in December 2023, incidence has become higher in women than men in younger and some middle-aged groups despite overall declines. In contrast, men still bear higher lifetime mortality [23]. Ethnic and ancestry differences are well documented: until December 2024 in the U.S., Black men had among the highest rates of lung cancer incidence and mortality, reflecting structural and access-related inequities, and East Asian ancestry is associated with a higher prevalence of actionable EGFR alterations in LUAD, which shapes therapy and outcomes; this information was made available in April 2025 [24,25].

Lung adenocarcinoma (LUAD) arises mainly from the distal lung epithelium. Recurrent oncogenic alterations drive it, most often in the RTK–RAS pathway (EGFR, KRAS including G12C, ALK, ROS1, MET exon-14 skipping, RET, BRAF, NTRK, ERBB2/HER2), together with tumor-suppressor losses (TP53, STK11, KEAP1) and lineage plasticity in alveolar type-II-like cell states. Whole-genome and single-cell studies now map these states and mutational processes with high resolution [17,26]. These genetics and cell-state programs shape immune evasion and variable response to therapy, with KEAP1/STK11 and related alterations linked to resistance to immune checkpoint blockade [27]. Worldwide, the most recent therapeutic approach matches treatment to genotype and stage: for EGFR-mutant disease, amivantamab plus lazertinib outperformed osimertinib in the first-line metastatic setting (October 2024), and amivantamab plus chemotherapy improved outcomes (November 2023) for EGFR exon-20 insertions [28,29]. In early-stage disease, adjuvant alectinib benefits resected ALK-positive NSCLC (April 2024), and adjuvant osimertinib improves overall survival (July 2023) in resected EGFR-mutant NSCLC [30,31]. Perioperative immunotherapy is now (since August 2023) standard for many resectable cases, with pembrolizumab improving survival. In a couple of studies published in August 2023 and June 2025, neoadjuvant nivolumab plus chemotherapy presented an overall survival benefit [32,33]. In addition, selective inhibitors (sotorasib, March 2023; adagrasib, June 2024) improve outcomes over docetaxel and continue to mature in phase 3 trials for KRAS-G12C LUAD [34,35]. Antibody–drug conjugates are expanding options, exemplified by trastuzumab deruxtecan activity in HER2-mutant NSCLC in January 2022 [36]. A pan-cancer proteogenomics study (published in 2024 at CELL) quantifies 2863 druggable proteins over 10 cancer types and analyzes mRNA–protein discordance across tumors. The study relates the amplification of copy numbers and mutations of EGFR with LUAD [37].

## 3. Gene Dysregulation in LUAD and Therapeutic Counteractions

**EGFR (Epidermal Growth Factor Receptor)** drives LUAD when its signaling becomes pathologically hyperactive in the distal lung epithelium. Activating kinase-domain mutations, which occur most commonly as exon 19 deletions or L858R, cause oncogene addiction that sustains proliferation and survival. Considering advanced EGFR-mutant NSCLC, the phase III FLAURA2 trial tested first-line osimertinib plus platinum–pemetrexed versus solo osimertinib in treatment-naïve [38]. This combination significantly prolonged survival without significant progression. In addition, it improved central nervous system control compared with monotherapy [38,39]. A final overall survival analysis reported in 2025 showed a statistically and clinically meaningful survival advantage for the combination, supporting intensified first-line therapy in appropriate patients.

**KRAS (Kirsten rat sarcoma viral oncogene homolog)** drives lung adenocarcinoma when mutations constitutively activate the RAS–MAPK pathway in distal lung epithelium. The canonical change is KRAS p.G12C, a smoking-associated variant that sustains proliferative and survival signaling and shapes tumor–immune interactions [40]. In 2025, Jänne et al. reported the largest first-line dataset testing a KRAS-G12C inhibitor with PD-(L)1 blockade, evaluating adagrasib plus pembrolizumab in advanced or metastatic KRAS-G12C NSCLC. The regimen showed encouraging antitumor activity across PD-L1 strata with a manageable safety profile and included the first survival readout for this combination strategy [41]. These results support a biology-consistent approach, direct KRAS-G12C inhibition paired with immunotherapy, to deepen responses in an oncogene-addicted subset, while ongoing trials refine selection and sequencing [42].

**ROS1 (ROS Proto-Oncogene 1, Receptor Tyrosine Kinase)** drives lung adenocarcinoma when ROS1 signaling is constitutively activated, promoting proliferation and survival [43]. The change leading to LUAD is a chromosomal rearrangement that creates ROS1 fusion kinases (for example, CD74–ROS1), establishing oncogene addiction, often in never- or light-smokers [44]. Early-generation ROS1 TKIs (crizotinib, entrectinib) are active but limited by intracranial control and by solvent-front resistance, such as G2032R [43]. In the phase 1/2 TRIDENT-1 study (published in January 2024), repotrectinib, which is a next-generation ROS1 inhibitor with activity against G2032R, produced durable responses in ROS1-fusion-positive NSCLC regardless of prior ROS1-TKI exposure [45]. Adverse events were mainly low grade and compatible with long-term treatment, and intracranial activity was observed, addressing key unmet needs in this subset [44,45].

**RET (Rearranged During Transfection proto-oncogene)** drives LUAD when aberrant RET kinase signaling becomes constitutively active, promoting proliferation and survival. The causal change is a RET gene fusion (for example, KIF5B–RET) that creates an oncogenic fusion kinase and oncogene addiction in distal lung epithelium. LIBRETTO-001 is an open-label phase I/II, single-arm study of selpercatinib in RET-dependent cancers; the 2025 final analysis reports efficacy and safety in RET-fusion-positive NSCLC. Objective response rate was 62% in previously platinum-treated patients (n = 247) and 83% in treatment-naïve patients (n = 69). Median duration of response was 31.6 months (pre-treated) and 20.3 months (treatment-naïve) at ~38 months’ follow-up; median progression-free survival was 26.2 and 22.0 months at ~40 months’ follow-up. Median overall survival reached 47.6 months in the pre-treated cohort and was not reached in the treatment-naïve cohort at ~43 months’ follow-up; adverse events were mainly low grade and compatible with long-term therapy [46].

**MET (Mesenchymal–Epithelial Transition proto-oncogene)** drives LUAD when exon 14 skipping removes the CBL docking site, stabilizing MET and sustaining oncogenic signaling; amplification can also act as a driver [47]. In GEOMETRY mono-1, capmatinib showed meaningful activity in METex14-positive NSCLC: ORR 68% in treatment-naïve (41/60) and 44% in previously treated (44/100) patients, with long follow-up (~46–67 months) and manageable safety. These data support capmatinib as a frontline option for METex14-positive NSCLC (NSCLC-wide efficacy; applies to LUAD subset) [48].

**ERBB2 (HER2)** drives LUAD via constitutive signaling, most often from exon 20 insertions; amplification/overexpression also occurs. He et al. reported a HER2 exon20ins stage IV NSCLC case with marked regression and 21 months without progression on trastuzumab deruxtecan [49]. Also, these observations align with trials showing robust T-DXd activity in HER2-mutant NSCLC (NSCLC-wide; LUAD subset included) [36,50].

**BRAF (B-Raf proto-oncogene, serine/threonine kinase)** drives LUAD when V600 substitutions, most often V600E, constitutively activate the MAPK pathway, sustaining mitogen-independent proliferation and survival. Swalduz et al. analyzed the French IFCT-2004 BLaDE cohort (163 evaluable). They found real-world dabrafenib plus trametinib (D-T) produced effective and tolerable outcomes across first- and later-line settings, mirroring clinical trials, and the results were published in January 2025. These real-world data are consistent with established first-line D-T and routine inclusion of BRAF testing in initial molecular panels, while the best sequencing with immunotherapy remains unresolved [51].

**NTRK1/2/3 (Neurotrophic Tyrosine Receptor Kinase 1/2/3; TRK) fusions** drive LUAD by constitutive TRK kinase signaling; the causal change is a chromosomal rearrangement creating an oncogenic TRK fusion, a rare event often in never-smokers. Cho et al. showed entrectinib produced clinically meaningful systemic and intracranial responses with manageable safety in NTRK-fusion-positive NSCLC, including patients with baseline brain metastases, supporting first-line use (NSCLC-wide; rare in LUAD). These data justify routine testing for TRK fusions and highlight the need for studies on long-term outcomes in this rare subset [52].

**NRG1 (Neuregulin-1)** drives LUAD when gene fusions create chimeric ligands that hyperactivate ERBB3/ERBB2 signaling, sustaining proliferation and survival. The causal change is an NRG1 fusion (e.g., CD74–NRG1), a rare event enriched in KRAS-wild-type, often mucinous, adenocarcinoma. In the phase II TAPUR basket trial, afatinib showed encouraging activity with durable disease control in NRG1-fusion tumors, warranting further study (basket trial; cross-indication) [53].

**PIK3CA (Phosphatidylinositol-4,5-bisphosphate 3-Kinase Catalytic subunit α)** drives LUAD when the PI3K–AKT–mTOR pathway is hyperactivated, promoting growth, survival, and drug tolerance. The key changes are activating PIK3CA mutations (e.g., E545K, H1047R) or upstream signals that sustain PI3K activity and blunt responses to single-agent therapies [54]. Preclinically, Yong Shim et al. showed that alpelisib combined with an autophagy inhibitor yields synergistic antitumor effects in PIK3CA-mutant NSCLC in vitro and in vivo, motivating a planned phase I trial [55].

**DDR2 (Discoidin Domain Receptor tyrosine kinase 2)** is a collagen-activated RTK whose dysregulation promotes EMT and engages PI3K/AKT and RAS/MEK/ERK signaling; evidence is strongest in lung squamous carcinoma and occasionally in adenocarcinoma. In addition, kinase-domain mutations or gene fusions can keep DDR2 active in the absence of collagen. Therefore, it leads to sustaining downstream signaling and increasing matrix metalloproteinases, which promote invasion. Indeed, DDR2 cooperates with integrins at the cell–matrix interface, stabilizing EMT programs and enhancing motility through cytoskeletal remodeling [56,57]. Oncogenic change mainly consists of somatic DDR2 mutations or fusions that drive constitutive signaling; mutation rates in LSCC range from 0% to ~4.6% with ethnic variation, and some cohorts show reduced DDR2 mRNA versus normal lungs [56,58]. Dasatinib inhibits DDR2-mutant cells, but clinical use is limited by toxicity and pathway complexity; resistance can arise via DDR2 T654I or NF1 loss [57,59].

**FGFR2/FGFR3 (Fibroblast Growth Factor Receptors 2/3)** drive cancer when fusions or activating mutations hyperactivate FGFR signaling. In NSCLC, FGFR fusions can lead to resistance to EGFR TKIs. FGFR2/3 fusions drive ligand-independent dimerization and sustained MAPK and PI3K signaling. Sometimes it emerges as a bypass track after EGFR inhibition.

Haura et al. added erdafitinib (8 mg qd) to ongoing osimertinib (80 mg qd) in an FGFR-fusion patient. It yielded rapid symptom relief and a CT-confirmed partial response by day 36 with manageable AEs (grade 2 hyperphosphatemia, mild dryness), and pan-cancer FGFR activity was noted (~21% PR, 21% SD). The FGFR TKI add-on in FGFR-fusion NSCLC while continuing osimertinib justifies prospective trials [60].

**MAP2K1 (mitogen-activated protein kinase kinase 1)** most often acts as an acquired resistance driver in LUAD; the K57N mutation hyperactivates MEK–ERK signaling and bypasses upstream blockade. Tan et al. reported (April 2024) a patient with EGFR-mutant, BRAF V600E NSCLC who developed acquired MAP2K1 K57N after EGFR/BRAF/MEK therapy; the combination with furmonertinib showed clinical activity before resistance emerged. High-dose furmonertinib monotherapy then failed, highlighting the need to monitor MAP2K1 K57N and to refine strategies for multi-targeted therapy-resistant disease [61].

**RIT1 (RAS-like without CAAX 1)** can drive LUAD when activating mutations, especially M90I, constitutively engage MAPK and PI3K signaling, promoting proliferation and therapeutic resistance. DiMarco et al. (July 2025) built the first RIT1 M90I mouse model that formed adenocarcinomas resembling human disease and showed RIT1-mutant cells are vulnerable to inhibitors of MAPK, PI3K, and cholesterol biosynthesis. SHP2 inhibitor migoprotafib combined with MAPK-pathway drugs suppressed growth and reversed RIT1-mediated resistance to the KRAS G12C inhibitor divarasib, supporting SHP2-based combinations [62].

**MYC (MYC proto-oncogene, basic helix–loop–helix transcription factor)** drives LUAD when amplification or upstream dysregulation elevates MYC activity, sustaining proliferation and survival. Because direct MYC inhibition is difficult, one approach blocks MYC-inducing Hippo signaling: first-in-class small molecules disrupt YAP–TEAD transcriptional activity [63]. Another approach uses molecular-glue degraders to recruit CRBN and degrade GSPT1, yielding selective cytotoxicity in neuroendocrine and related tumors and suggesting refined strategies for aggressive cancers [64].

**YAP1 (Yes-Associated Protein 1)** drives LUAD when Hippo signaling is inactivated (e.g., NF2 loss or YAP/TAZ fusions), enabling YAP/TAZ–TEAD-dependent transcription that sustains proliferation and survival. Chapeau et al. report IAG933, a potent, selective small-molecule disruptor of the YAP/TAZ–TEAD interaction with strong preclinical antitumor activity, good tolerability, and favorable pharmacokinetics, supporting a first-in-human trial [65]. Related phase-1 programs include TEAD lipid-pocket inhibitors VT3989 (April 2023, NCT04665206) and IK-930 (June 2022, NCT05228015), with early clinical reports in conference abstracts [63,66].

**NFE2L2 (Nuclear Factor Erythroid 2 2-related factor 2; NRF2)** drives LUAD when the KEAP1–NRF2 axis is disrupted (KEAP1 loss or NFE2L2 activation), causing constitutive antioxidant programs and drug tolerance. Galan-Cobo et al. show this disruption compensatorily engages ATR–CHK1, creating sensitivity to ATR inhibitors; in KEAP1 and/or LKB1-deficient NSCLC models, ceralasertib was strongly active, synergized with gemcitabine, and enhanced anti-tumor immunity. Consistently, the phase-II HUDSON trial (August 2025) found greater benefit from ceralasertib plus durvalumab in LKB1/KEAP1-deficient patients, supporting ATRi-based combinations and biomarker-guided selection [67].

**TP53 (Tumor Protein p53)** safeguards genome integrity; in LUAD, loss-of-function TP53 mutations disable DNA-damage responses and apoptosis, promoting malignant progression [68]. In a phase I, multicenter study (NCT04383938), eprenetapopt (APR-246) plus pembrolizumab showed acceptable safety and signs of clinical activity, including responses in NSCLC. These data support testing p53 reactivation with PD-1 blockade as a rational strategy for TP53-defective tumors (NSCLC cohort; cross-indication to LUAD) [69].

**STK11 (serine/threonine kinase 11; LKB1)** is a tumor suppressor; its loss in NSCLC disrupts AMPK signaling, rewires metabolism, and correlates with poor response to immunotherapy [70]. Feng et al. highlight frequent STK11 mutation without a defined first-line standard and report prolonged PFS with first-line cadonilimab plus pemetrexed/carboplatin in an STK11-mutant case. These findings support evaluating chemo–immunotherapy and personalized strategies for STK11-mutant NSCLC in prospective studies [71].

**NF1 (neurofibromin 1)** is an RAS-GAP; loss-of-function removes negative control of RAS, hyperactivating MAPK/PI3K signaling in NF1-associated tumors [72]. In adults with symptomatic, inoperable plexiform neurofibromas, selumetinib showed objective responses in a phase 2 trial and improved outcomes over placebo in the randomized KOMET phase 3 study (non-lung tumor context; cross-indication) [72,73]. Moreover, preclinical work identifies autophagy inhibition as a synthetic lethal vulnerability and shows SHP2 inhibition plus hydroxychloroquine suppresses NF1-MPNST growth, nominating combination strategies [74,75].

**PTEN (Phosphatase and Tensin homolog) loss-of-function** derepresses PI3K–AKT–mTOR signaling and impairs DNA-damage responses, promoting LUAD/NSCLC; mutations or silencing that reduce PTEN activity are the key changes. Dunne et al. engineered PTEN-knockdown H460/A549 cells. They showed that ceralasertib (ATR inhibitor) plus radiotherapy reduced clonogenic survival, delayed DNA repair, and suppressed PTEN-deficient xenograft growth versus PTEN-proficient controls. In pneumonitis-prone mice, the combination did not meaningfully worsen early inflammatory readouts (aside from a week-4 macrophage rise), supporting ATRi + RT testing in PTEN-mutant NSCLC (NSCLC models; not LUAD-restricted) [76].

**CDKN2A (Cyclin-dependent kinase inhibitor 2A)** encodes p16^INK4A^/p14^ARF^; its loss (deletion/mutation or methylation) deregulates the cyclin-D–CDK4/6–RB and p53 pathways, enabling unchecked proliferation in LUAD. Lv et al. review NSCLC cell-cycle dysregulation and report that abnormalities in the cyclin-D–CDK4/6–INK4–RB pathway are common, with preclinical and clinical studies showing encouraging activity of CDK4/6 inhibitors alone or in combinations (pan-NSCLC review; LUAD extrapolation). These data support testing CDK4/6 blockade, particularly in tumors with CDKN2A loss or RB-intact disease, within biomarker-driven trials [77].

**RB1 (RB transcriptional corepressor 1)** restrains G1–S transition by repressing E2F; its loss in lung cancer induces proliferation, promotes genomic instability, and fuels aggressive behavior. The causal change is biallelic inactivation (mutation/deletion or functional loss), which disables the RB pathway and limits benefit from CDK4/6-directed strategies. Huang et al. summarize challenges and emerging tactics for RB1-deficient tumors, synthetic lethality, replication-stress exploitation, and lineage vulnerabilities, supporting personalized therapy development [78].

**SMARCA4/BRG1** loss disables SWI/SNF chromatin remodeling in LUAD and correlates with dedifferentiation and poor prognosis [79]. Loss-of-function mutations can elevate GLI1 activity (sometimes independent of canonical Hedgehog), and high GLI1 is associated with aggressive NSCLC biology [80,81]. These data support testing GLI1-directed strategies for BRG1-deficient LUAD, with preclinical and translational evidence motivating GLI1 blockade and combinations [82].

**KEAP1 (Kelch-like ECH-associated protein 1)** restrains NRF2; KEAP1 loss or mutation constitutively activates NRF2 programs, driving redox adaptation, metabolic rewiring, and therapy resistance in NSCLC. KRAS-mutant tumors with co-mutant STK11/LKB1 and KEAP1 rely on ATR–CHK1 signaling; the ATR inhibitor ceralasertib shows strong preclinical activity and enhances antitumor immunity. Ceralasertib plus durvalumab produced greater benefit in LKB1/KEAP1-deficient patients and is now in phase III testing. However, this is hypothesis-generating for ATRi-based combinations and biomarker selection, pending phase-3 confirmation [67].

**RNF115 (Ring Finger Protein 115)** encodes an E3 ubiquitin ligase; its dysregulation promotes oncogenic signaling, proliferation, and invasion in lung adenocarcinoma. The relevant change is overexpression (often with copy-number gain), which is associated with aggressive clinicopathologic features. Wu et al. showed that high RNF115 expression predicts poorer prognosis and is linked to oncogenic behavior in LUAD, nominating RNF115 as a biomarker and potential target. Independent validation and tractability studies are required [83].

**CDH1 (Cadherin-1)** encodes E-cadherin; reduced expression, via promoter hypermethylation or mutation, weakens adherens junctions and promotes EMT, invasion, and metastasis in NSCLC. Loss of E-cadherin weakens cell–cell adhesion and releases β-catenin, which facilitates EMT, invasion, and metastasis. Sarne et al. created pyrosequencing assays for CDH1, CDKN2A, RASSF1A, TERT, and WT1; in 144 FFPE NSCLC samples, significant hypermethylation was observed for TERT and WT1 (not consistently for CDH1), with supportive cell-line studies. They conclude these promoter-methylation markers have diagnostic (and possible predictive) value, warranting deeper protein/pathway and drug-response analyses [84].

**CHEK2 (Checkpoint Kinase 2)** encodes a DDR kinase (CHK2); its loss can raise tumor mutational burden and activate cGAS–STING, while high CHEK2 expression has been linked to weaker benefit from immune checkpoint inhibitors. A January 2025 review synthesizes these data and proposes combining CHK1/2 inhibition with ICIs as a rational strategy [85]. This remains hypothesis-generating without randomized clinical data. Complementing this, an April 2024 Cancer Letters study shows CHEK2 deficiency increases PD-1 response with higher CD8^+^ T-cell infiltration in murine models [86].

**ERBB3 (Erb-B2 receptor tyrosine kinase 3)** facilitates PI3K/AKT signaling downstream of mutant EGFR, leading to the disturbed function of PI3K activation via adaptor proteins. At the same time, EGFR-activating mutations drive LUAD. In inducible EGFR^L858R^ models, deleting Erbb3 at induction did not block tumor initiation or EGFR-TKI sensitivity. Still, acute Erbb3 loss later curtailed growth of established tumors despite persistent AKT/ERK via GAB1/2 and compensatory ERBB2/MET phosphorylation. The study claims that ERBB3, as a context-dependent facilitator rather than a universal dependency, can be considered a combination strategy that co-targets EGFR and parallel adapters/RTKs [87].

**FOXA1 (Forkhead Box A1)** can promote LUAD by directly activating the HER2 promoter, elevating HER2/PI3K/AKT signaling, and supporting proliferation and survival. Zhao et al. showed that disitamab vedotin (RC48) exerts antitumor effects in lung cancer cells by targeting both the HER2/PI3K/AKT and FOXA1/HER2/PI3K/AKT axes, with higher FOXA1 and HER2 correlating with worse prognosis [88]. Real-world and clinical reports further support RC48 activity in HER2-altered NSCLC, reinforcing FOXA1–HER2 as a therapeutically relevant circuit [89].

**IDH1 (Isocitrate Dehydrogenase 1)** mutations are rare in LUAD (~0.5%) but create a neomorphic enzyme that produces D-2-hydroxyglutarate, driving epigenetic reprogramming and tumor progression [90,91]. In LUAD, IDH1/2 mutations often appear in high-grade tumors, co-occur with KRAS, and behave as subclonal/branching drivers with uncertain standalone clinical impact [91,92]. While IDH1 inhibitors are effective in other cancers, LUAD evidence remains limited; ongoing work in solid tumors supports selective, trial-based evaluation in IDH1-mutant NSCLC [93].

**IDH2 (Isocitrate Dehydrogenase 2)** supports LUAD by sustaining redox balance; high wild-type IDH2 expression correlates with poorer survival in chemotherapy-treated patients. Lentiviral knockdown or pharmacologic inhibition increased cisplatin and radiation sensitivity, resensitized cisplatin-resistant cells, and elevated ROS without major OCR/ECAR changes. In xenografts, IDH2 silencing enhanced the antitumor effect of cisplatin, supporting IDH2 inhibition as an adjunct in NSCLC therapy [94].

**PIK3R1 (Phosphoinositide-3-Kinase Regulatory Subunit 1, p85α)** restrains PI3K signaling; in LUAD, elevated miR-21-5p down-regulates PIK3R1, releasing PI3K/AKT activity and promoting progression. Du et al. showed that miR-21-5p is upregulated in LUAD, inversely correlates with PIK3R1, and predicts poorer survival, nominating the miR-21-5p/PIK3R1 axis as a prognostic biomarker [95]. The results align with the central role of the PI3K/AKT/mTOR pathway in NSCLC and support therapeutic strategies that restore PIK3R1 function or inhibit downstream signaling in LUAD preclinical and clinical studies [96].

**PTPRT (Protein Tyrosine Phosphatase, Receptor Type T)** is a transmembrane phosphatase; its downregulation in LUAD elevates survivin (BIRC5) and drives proliferation, migration, and invasion [97]. PTPRT loss also shapes immunotherapy response by activating cGAS–STING signaling, increasing interferon programs and immune infiltration, and predicting greater benefit from anti-PD-1/PD-1/PD-L1 therapy [98]. These data nominate PTPRT as a biomarker for prognosis and for selecting or combining immune checkpoint inhibitors in LUAD. Prospective LUAD-specific validation is needed before using it to choose or combine ICIs.

**PPFIBP1 (liprin-β1)** is a scaffolding protein that organizes adhesion/signaling complexes. Its dysregulation promotes motility and invasion in cancer. It scaffolds LAR-family phosphatases at adhesion sites and coordinates integrin-linked signaling that supports motility [99]. PPFIBP1–ALK fusions identified in pulmonary inflammatory myofibroblastic tumors, which underscores the relevance of PPFIBP1 in disease [100]. Moreover, the upregulation of the related axis gene PPP1R3G was recognized in LUAD and predicts poor survival with immune-infiltration features [101].

**U2AF1 (U2 Small Nuclear RNA Auxiliary Factor 1)** is a splicing factor; the recurrent S34F mutation alters 3′ splice-site choice, reshapes LUAD programs (e.g., EMT/mitotic stress), and can aid malignant progression [102]. These data suggest U2AF1^S34F^ grants stress tolerance that facilitates KRAS-driven transformation, implying context-specific vulnerabilities for combination therapy in LUAD. Overall, potential combination-therapy vulnerabilities are hypothesis-generating and need functional and clinical testing [103].

**NKX2-1 (NK2 Homeobox 1; TTF-1)** is a lineage transcription factor frequently amplified or overexpressed in LUAD; such gain sustains epithelial identity and tumor fitness. TTF-1 maintains alveolar type-II lineage programs and surfactant genes; amplification or overexpression creates lineage dependency that supports tumor fitness. Recent work shows lineage TF perturbation and enhancer re-wiring as exploitable vulnerabilities in TTF-1–addicted tumors [104].

**RBM10 (RNA Binding Motif Protein 10)** is a splicing regulator whose loss-of-function alters exon usage, promotes LUAD growth, and can confer drug resistance; RBM10-altered cells reprogram PI3K/AKT/MAPK signaling via aberrant splicing. These features flag RBM10 as a therapeutically relevant tumor suppressor in LUAD [105].

**SETD2 (SET Domain–Containing 2)** H3K36me3 methyltransferase; SETD2 loss accelerates KRAS-driven LUAD and creates dependencies on oxidative phosphorylation and mTORC1. SETD2 deficiency may nominate patients for OXPHOS/mTORC1-targeted strategies [106].

**ARID1A/KMT2D/KMT2C (Chromatin remodeling and enhancer modifiers)** drives recurrent alterations in NSCLC that reshape enhancer usage and transcriptional programs, fostering LUAD progression and therapy resistance. Indeed, these epigenetic lesions point up chromatin-targeted opportunities. Contemporary reviews underscore their relevance in LUAD biology [107].

**ATM (Ataxia-Telangiectasia Mutated)** is the most frequently mutated DDR gene in LUAD. Its loss compromises double-strand break signaling through the ATM–CHK2–p53 axis, shaping sensitivity to DNA-damage response combinations. ATM-mutant tumors show distinct co-mutation patterns (e.g., KRAS) and potential therapeutic vulnerabilities, with implications for ICI responses. These data support ATM as a stratification biomarker [108].

**PTPRD (Protein Tyrosine Phosphatase Receptor-Type D)** is a broadly inactivated phosphatase/tumor suppressor; multi-cancer screening and real-world datasets identify PTPRD mutation as a recurrent event with clinical impact, including in LUAD. PTPRD restrains growth signaling by dephosphorylating substrates such as STAT3; its loss sustains STAT3 activity and may affect response to targeted and immune therapies. Moreover, its loss may shape progression and therapy response [109].

**MGA (MAX Gene Associated)** as a MYC-network repressor is recurrently inactivated in LUAD, enriched in smokers, and often co-mutant with KRAS. This gene partners with MAX to repress MYC; loss of MGA derepresses MYC programs and cooperates with KRAS to accelerate disease progression. Functional data support an oncogenic cooperation that accelerates disease. This positions MGA loss as a meaningful LUAD driver [110].

**ERBB4 (Erb-B2 Receptor Tyrosine Kinase 4)** rarely participates in events, including EGFR::ERBB4 fusions, in NSCLC (inference to LUAD). It forms active heterodimers with other ERBB receptors, activating PI3K/AKT and MAPK cascades; fusions can create constitutively active kinases. A recent case showed durable radiographic benefit on sequential EGFR TKIs, underscoring the actionability of ERBB-family fusions in NSCLC (basket; cross-indication) [111].

**AKT1 (AKT Serine/Threonine Kinase 1; E17K)** is a hotspot mutation that activates PI3K/AKT signaling across cancers and exists in NSCLC subsets; early clinical data with AKT inhibitors (e.g., capivasertib). The E17K mutation increases AKT1 membrane binding, resulting in the constitutive activation of downstream survival and growth signaling pathways. This gene support mutation-directed targeting, informing PI3K/AKT-centric LUAD (basket; cross-indication) trials [112].

**NRAS/HRAS (Neuroblastoma/Harvey Rat Sarcoma Viral Oncogenes)** Non-KRAS RAS mutations are uncommon LUAD (pan-NSCLC) drivers but clinically relevant when present. Also, activating NRAS/HRAS mutations engages the MAPK and PI3K pathways. Modern NSCLC overviews emphasize their biology, detection, and emerging targeted strategies [113].

**CTNNB1/APC (β-Catenin/Adenomatous Polyposis Coli; WNT Pathway)** affects WNT/β-catenin signaling via CTNNB1 activation or APC loss, promoting LUAD (pan-NSCLC) progression and immune evasion; recent reviews in NSCLC outline therapeutic avenues to block this axis [114].

**TSC1/TSC2 (Tuberous Sclerosis Complex 1/2)** hyperactivates mTORC1 and may influence LUAD immunogenicity when lost. Loss of TSC1/2 activates RHEB and mTORC1, altering metabolism and immune context and potentially influencing ICI sensitivity. Pan-cancer (pan-cancer/NSCLC models; cross-indication) evidence suggests enhanced ICI sensitivity with TSC1/2 alterations, informing mTOR- and ICI-based strategies [115].

**MDM2 (Mouse Double Minute 2)** dampens p53 and has been linked to hyper-progression on ICIs when amplified or overexpressed. New reviews also discuss combining MDM2 inhibitors with immunotherapy as a rational, testable approach in NSCLC (cross-indication; not LUAD-specific) [116].

**CCNE1 (Cyclin E1)** drives cell-cycle acceleration and genomic instability when amplified. CCNE1 amplification drives CDK2-dependent S-phase entry and replication stress, nominating CDK2 or replication-stress–focused strategies. Emerging oncology literature (including 2025 perspectives) frames CCNE1 as a prognostic and therapeutic biomarker, guiding trials of CDK/microtubule-directed regimens (cross-indication; ovarian-anchored evidence) [117].

**TERT/TERC (Telomerase Reverse Transcriptase/RNA Component)**: Telomerase activation in NSCLC arises via promoter mutations and/or amplification, with liquid-biopsy TERT showing diagnostic/prognostic value. Meanwhile, TERT promoter mutations create de novo ETS-binding motifs that increase TERT transcription and enable telomerase-mediated immortalization. Recent mechanistic work clarifies atypical promoter events (NSCLC-wide; not LUAD-specific) [118,119].

**KMT2D/KMT2C (Lysine Methyltransferase 2D/2C; MLL4/MLL3):** Enhancer-modifying tumor suppressors whose loss rewires chromatin and can shape LUAD biology. In lung cancer, KMT2D deficiency impairs super-enhancers and creates glycolytic and RTK/RAS dependencies, while KMT2C loss in high-grade fetal-type LUAD correlates with reduced homologous-recombination factors and potential PARP-inhibitor sensitivity (NSCLC subsets; extrapolated to LUAD) [120,121,122].

Table 3 summarizes all genes we described that drive LUAD, grouping them by Role and concatenating their symbols alongside the primary pathways they influence. It aggregates pathway scores and resistance tendencies, highlighting dominant axes such as RTK/RAS/MAPK, PI3K/AKT/mTOR, cell-cycle/RB, DDR, chromatin/epigenetic, and redox/NRF2.

## 4. Review on Drug Repurposing in LUAD

### 4.1. Connectivity Map/LINCS

Connectivity Map (CMap) and the NIH LINCS L1000 resource operationalize “signature reversal”: drugs whose perturbational transcriptomes most strongly invert a disease gene-expression signature are prioritized as candidates. In LUAD, multiple studies build subtype/risk signatures and query CMap to surface small molecules predicted to counteract malignant programs (e.g., withaferin A, everolimus, saracatinib) or to target high-risk groups, demonstrating practical, disease-specific use [123,124,125]. Methodologically, the original CMap (microarray profiles across fewer cell lines/compounds) established the concept and direct full-transcriptome matching. At the same time, LINCS/L1000 scaled the approach > 1000-fold via a reduced-representation assay (978 “landmark” genes with imputed remainder), enabling millions of dose/time/cell-context profiles, now accessible via CLUE for programmatic analysis. The trade-off is clear: CMap’s smaller, full-array data are simple to interpret but limited in coverage. In addition, L1000 brings massive breadth, richer perturbational context (including genetic perturbations). Moreover, it presents a better statistical power for connectivity scoring, at the cost of relying on inferred genes and being sensitive to cell-line context [126,127]. Importantly, large-scale evaluations show that stronger signature reversion is associated with greater antitumor efficacy, and in lung models, the L1000 assay has been profiled head-to-head with Cell Painting in A549 cells. It also underscores complementary readouts for mechanism mapping and repurposing triage [128,129].

### 4.2. Network Medicine/Interactome Proximity

Network medicine models disease as a perturbed “module” in the human protein–protein interactome and scores candidate drugs by interactome proximity, the network distance between a drug’s targets and disease proteins. Therefore, closer drugs are more likely to modulate the causal neighborhood. Foundational work established and validated proximity at the population scale, and generalized it into a practical repurposing framework and platform tools [3,8]. In LUAD, integrative network analyses identified actionable modules, most prominently Aurora kinase–centered programs in early-stage invasive LUAD with functional validation. In addition, LUAD-specific pipelines have combined target-gene prediction with network distance/overlap to prioritize repurposable drugs [130,131]. Method variants include patient-specific module construction (GPSnet) and alternative graph metrics (e.g., minimum/mean/median distances, diffusion). Also, benchmarking studies comparing their behavior and performance were conducted [132,133]. Advantages vs. signature-based (CMap/LINCS) screening: proximity is cell-line–agnostic, mechanism-grounded, readily integrates multi-omics and genetics, and supports patient-level personalization via disease modules. On the other hand, trade-offs depend on interactome completeness/target annotation and typically lack directionality or dose/time information that transcriptional signatures encode. Comprehensive recent reviews position network medicine as a scalable, interpretable virtual screening layer that complements expression-based and learning-based approaches [134].

### 4.3. Knowledge-Graph (KG) Methods

Knowledge-graph (KG) methods encode drugs, diseases, genes, pathways, side-effects, and more as a multi-relational graph, then learn or search over its topology to predict plausible drug–disease links. Early, influential work (Hetionet/Project Rephetio) showed that metapaths traversing curated biomedical edges can prioritize repurposing candidates in a transparent, testable way, establishing the paradigm [9]. Recent systems scale this idea with graph learning: TxGNN (Nature Medicine, 2024) is a graph foundation model that performs zero-shot indication and contraindication prediction across ~17k diseases. The model enables generalization to under-studied conditions. DREAMwalk improves link prediction by generating semantically guided multi-layer random walks, while XG4Repo emphasizes explainable path evidence for each prediction [135]. For LUAD specifically, KG-driven pipelines using graph attention networks have been used to nominate LUAD target genes and then map drugs by gene overlap/network distance. This pipeline illustrates disease-focused applicability. Newer resources such as TarKG strengthen KG coverage for target discovery and downstream repurposing [130,136]. Compared with signature-based approaches (CMap/LINCS) or interactome-proximity alone, KG methods natively integrate heterogeneous evidence (omics, literature, clinical phenotypes). Although these methods support multi-hop mechanistic reasoning and zero-shot transfer, and can provide human-readable rationales via metapaths, they inherit biases and incompleteness from source databases. They can be sensitive to graph construction choices and edge quality [137]. Another study (Nature Biomedical Engineering, July 2025) presents an interpretable transformer-based graph model that predicts cancer genes by integrating multi-omics data with biological network structure. It provides explanations at both the feature and network levels and generalizes across datasets [138].

### 4.4. Ligand-Based Similarity/Chemogenomics

Ligand-based similarity and chemogenomics repurpose drugs by exploiting the “similar ligands to similar targets/effects” principle: from classic 2D fingerprint approaches, like the Similarity Ensemble Approach (SEA) that infer off-targets/indications directly from structural resemblance, to modern chemogenomic drug–target interaction (DTI) models that learn from large bioactivity matrices. In practice, first, LUAD studies often use ligand-based target prediction (e.g., SwissTargetPrediction) to nominate targets for candidate compounds. Then, they validate these predictions with docking/wet-lab assays in A549/H1299 models. Recent examples include afzelin (NQO2-linked) and 6-methoxydihydrosanguinarine pipelines, with similar LUAD workflows reported for other small molecules [10,139,140]. Compared with structure-based screening, ligand-based methods are fast, scalable, and surprisingly effective for polypharmacology and scaffold hopping (especially when augmented by richer bioactivity signatures like the Chemical Checker). However, they can suffer from activity cliffs and cell-context ambiguity unless anchored by orthogonal evidence [141]. Within this family, simple 2D similarity (ECFP/Tanimoto) is highly interpretable and strong on close analogs. At the same time, chemogenomic DTI methods (e.g., matrix-factorization variants) generalize better across sparse data by pooling signals across drugs and targets. But they depend on the breadth/quality of existing interaction data [142]. Hybrid schemes that bridge ligand- and structure-based views (e.g., interaction fingerprints like PLEC/FIFI) often boost early enrichment and help explain predictions. Also, they offer a practical compromise for LUAD pipelines that combine target prediction, then docking, and finally experimental triage [143].

### 4.5. Side-Effect (Phenotypic) Similarity Mining

Side-effect (phenotypic) similarity mining repurposes drugs by assuming that compounds causing similar clinical phenotypes (ADRs) are likely to share targets or mechanisms. Thus, to infer new indications, therapeutic potential clusters drugs by adverse-event profiles from resources like SIDER and newer curated datasets (e.g., OnSIDES). Foundational work showed that phenotypic side-effect resemblance alone can recover shared targets across chemically dissimilar drugs. It establishes a transparent, metapath-like rationale for repositioning. Modern pipelines extend this with pharmacovigilance signal processing (FAERS) and reporting standards (READUS-PV) to curb bias [11,144,145]. Compared with CMap/LINCS (expression reversal) and interactome proximity (mechanistic network distance), side-effect mining is label-free, inexpensive, and directly grounded in human clinical phenotypes, which helps capture real-world polypharmacology and complements omics-first screens. Compared with KG/graph methods, it provides human-interpretable evidence (shared ADR patterns) without heavy model training. Its trade-offs are important: spontaneous reporting and indication confounding can create spurious “inverse signals,” and disease specificity is lower than transcriptomic or network module approaches. In addition, directionality (benefit vs. harm) often needs external triangulation (e.g., EHR target-trial emulation or LINCS signature checks) before LUAD-focused follow-up. Recent reviews codify FAERS-based repurposing strategies (including inverse-signal analyses) and best practices for disproportionality analyses. These studies suggest phenotypic mining as a pragmatic first pass that should be integrated with LUAD molecular evidence rather than used in isolation [4].

### 4.6. Structure-Based Virtual Screening & Inverse Docking

Structure-based virtual screening (SBVS) and inverse docking repurpose drugs by modeling how approved molecules bind LUAD-relevant targets. Conversely, by docking one drug across many human proteins to hypothesize its mechanism. In LUAD specifically, recent pipelines use docking as the triage step on omics-derived hits: an OMICS 2024 study combined ML with transcriptomics, then used docking to prioritize candidates (e.g., HSP90 inhibitors, cardiac glycosides, trifluoperazine) for LUAD. Another LUAD paper targeted the TPX2–AURKA interface and predicted the approved TKI dacomitinib as a plausible PPI disruptor. Also, network pharmacology + docking studies have nominated active components for LUAD herbal preparations; more recently, docking/MD supported telmisartan and pioglitazone as PPARG-modulating LUAD candidates with exosomal delivery concepts [146,147,148]. Methodologically, inverse-docking servers (e.g., PharmMapper reverse pharmacophore mapping and ReverseDock blind docking) aid target deconvolution for repurposed ligands. At the same time, next-gen SBVS improves pose/affinity accuracy via receptor flexibility and AI-accelerated rescoring (e.g., RosettaVS, 2024) and codified best practices [149,150,151,152,153].

### 4.7. Machine Learning/Deep Learning

Machine learning (ML) and deep learning (DL) repurposing pipelines for LUAD typically learn disease–drug relationships from high-dimensional omics and chemical features, then rank approved compounds for testing. LUAD-focused examples include a graph-attention pipeline that first predicts LUAD driver/marker genes from TCGA and then prioritizes drugs by gene overlap/network distance (yielding concrete candidates for LUAD follow-up). Also, an integrative ML + docking study that derived LUAD systems biomarkers and nominated tractable agents (e.g., HSP90 inhibitors, cardiac glycosides) [130,146]. Beyond LUAD only, NSCLC-specific DL that fuses transcriptomics + chemical structure recovered the antipsychotic pimozide and validated cytotoxicity in A549 cells. This study illustrates how multimodal DL can surface non-obvious oncology uses [154]. Methodologically, newer representation-learning approaches learn similarity directly from perturbational signatures (e.g., DrSim) instead of hand-crafted metrics. Moreover, graph foundation models (TxGNN) perform zero-shot indication/contraindication prediction across ~17k diseases. This approach is useful when LUAD labels are sparse [135,155]. Compared with signature-reversal (CMap/LINCS) and network-proximity alone, ML/DL can jointly model chemistry, pathways, and multi-omics, capture non-linear effects, and generalize better across data regimes. Trade-offs include dependence on training data quality (batch effects, label noise), potential domain shift between cell lines and LUAD tumors, and interpretability challenges. Hence, best practice is to pair ML/DL ranking with orthogonal evidence (e.g., docking, pathway context) before LUAD wet-lab validation. Recent surveys in oncology ML document these strengths/limits and dataset requirements (e.g., for drug–target/response modeling), helping set expectations for LUAD repurposing studies [156,157]. CHIEF (a pathology foundation model proposed in 2024) learns from whole-slide images with weak supervision. It combines unsupervised tile-level pretraining with weakly supervised slide-level pretraining. The results demonstrate strong diagnostic and prognostic transferability across various cancers [158].

By the way, ML/DL models for repurposing can overfit to batch effects, proxy labels, and cell-line artifacts, yielding inflated metrics that do not transfer to independent LUAD cohorts. Underfitting occurs when small or heterogeneous datasets, combined with strong regularization, fail to capture nonlinear biological processes. To improve interpretability and trust, pair rankings with feature- and pathway-level attributions (e.g., SHAP/Integrated Gradients and enrichment summaries) and report cross-cohort, time-split, and leakage-controlled validation.

### 4.8. Pathway- & Enrichment-Based Approaches

Pathway- and enrichment-based repurposing for LUAD starts by deriving disease signatures (e.g., DEGs or risk-strata genes) and mapping them to perturbed KEGG/GO/WGCNA pathways. Then, drugs are prioritized if their known pathway impacts oppose LUAD programs or if their mechanisms are enriched against dysregulated modules. A canonical LUAD study intersected 57 LUAD KEGG pathways with CMap compound–pathway effects, highlighted p53 signaling (hub genes CCNB1/CCNB2/CDK1/CDKN2A/CHEK1), and nominated agents such as ciclopirox and pyrvinium, with docking showing CHEK1 binding (daunorubicin, mycophenolic acid, pyrvinium) [159]. An immune-prognostic LUAD model used CMap to surface resveratrol, methotrexate, and phenoxybenzamine. Then, it validated A549 growth inhibition experimentally, illustrating a full enrichment, then CMap, and finally a wet-lab loop [124]. A separate LUAD prognostic/enrichment pipeline screened CMap hits and verified oxibendazole as anti-proliferative in LUAD cells [125]. At the NSCLC level (generalizable to LUAD cohorts), a WGCNA, TF–TG, and DGIdb enrichment workflow proposed 16 candidate drugs by aligning co-expression modules to drug–gene interactions [160]. Methodologically, Drug Mechanism Enrichment Analysis (DMEA) boosts prioritization by aggregating drugs into MOA sets for GSEA-like testing, useful when single-compound signals are noisy [161]. Best-practice reviews of signature-based repurposing (CMap/LINCS) help choose robust scoring and controls. In practice, enrichment hits are strongest when triangulated with orthogonal evidence (e.g., docking or network proximity) before LUAD wet-lab follow-up [162].

## 5. Conclusions

Computational and experimental drug repurposing offer a pragmatic path to accelerate therapies in lung adenocarcinoma. In this study, we investigated the most potent driver genes in LUAD and organized eight computational method families to link them to LUAD driver biology. We highlighted that regularly reviewing evidence, computational approaches, and phenotypic or clinical signals can enhance prioritization. Current opportunities are clustered in biomarker-defined subgroups and pathways (e.g., RTK/MAPK, PI3K, KRAS–STK11/KEAP1–NRF2, Hippo/TEAD, and DNA damage response), but evidence strength varies, and cross-indication findings should be labeled and validated. As the minimum requirement, a reproducible workflow should include curated datasets, pre-specified analyses, leakage and bias checks, open code, and docking or assay-level triage before small-scale prospective testing. Building shared benchmarks and conducting biomarker-selected, prospective studies will be essential to turn repurposing leads into clinical options for LUAD.

## Figures and Tables

**Table 1:** Experimental drug repurposing methods.

Method	Description	
Cell-based phenotypic screening (Cell Painting) [1]	Goal	Unbiased cellular assays; morphology/profiles cluster MoA
	Pros	Detects multi-target effects; no prior target needed
	Cons	Assay artifacts; translation gap; lab infrastructure
Phenotypic drug discovery across screening models [2]	Goal	Screen in complex models for efficacy/toxicity
	Pros	Higher physiological relevance; emergent effects
	Cons	Costly; low throughput; ethical constraints
EHR/claims mining and target-trial emulation [3]	Goal	Estimate the effects of existing drugs on new outcomes
	Pros	Real-world, human-level effects; diverse outcomes
	Cons	Confounding and bias; data access/cleaning burdens
Pharmacovigilance (FAERS) inverse-signal analyses [4]	Goal	Identify protective drug–outcome associations in safety data
	Pros	Cheap; wide coverage; early human signals
	Cons	Reporting/indication bias; noisy; weak causality
Human genetics for indication selection [5]	Goal	Align drug mechanisms with GWAS/OMIM evidence
	Pros	Higher clinical success; directionality clues
	Cons	Limited to genetically mediated disease; small effects
Drug-target Mendelian randomization (cis-MR) [6]	Goal	Use genetic instruments on targets to infer efficacy/safety
	Pros	Causal on-target prediction; dose–response hints
	Cons	Instrument validity/pleiotropy limits; target coverage

**Table 2:** Computational (in silico) drug repurposing methods.

Method	Description	
Connectivity Map/LINCS [7]	Goal	Match disease expression signatures to drugs that invert them
	Pros	Human-relevant; target-agnostic; scalable; novel hits
	Con	Cell-line mismatch; off-target confounding; signature quality dependent
Network medicine/interactome proximity [8]	Goal	Rank drugs whose targets lie near disease modules in the interactome
	Pros	Mechanistic context; polypharmacology; interpretable
	Cons	Incomplete or biased networks; target mapping gaps
Knowledge-graph (KG) methods [9]	Goal	Predict drug–disease links using heterogeneous biomedical graphs
	Pros	Integrates diverse evidence; handles indirect paths
	Cons	Data noise; edge bias; KG engineering required
Ligand-based similarity/chemogenomics [10]	Goal	Infer new targets/indications from chemical/bioactivity similarity
	Pros	Fast; simple; reveals off-targets
	Cons	Limited novelty; activity cliffs; needs high-quality assays
Side-effect (phenotypic) similarity mining [11]	Goal	Use shared adverse-event profiles to infer common targets/uses
	Pros	Human phenotype signal; orthogonal to chemistry
	Cons	Confounding/indication bias; under-reporting; rare events
Structure-based virtual screening & inverse docking [12]	Goal	Dock approved drugs against target panels to find binders
	Pros	Atomic mechanism; target-specific; repurpose to novel targets
	Cons	Scoring errors, protein flexibility; structure availability
Machine learning/deep learning [13,14]	Goal	Learn patterns across chemical, target, disease features
	Pros	Captures nonlinear patterns; scalable
	Cons	Black-box; data leakage risk; needs large labeled sets
Pathway- and enrichment-based [15]	Goal	Prioritize drugs that modulate disease-enriched pathways
	Pros	Interpretable; mechanism-level view
	Cons	Pathway incompleteness; over-representation bias

**Table 3:** Summarization of genes and related pathways in LUAD.

Role		Genes	Pathways
Co-receptor		ERBB3	PI3K/AKT/mTOR, RTK/RAS/MAPK
Lineage TF		FOXA1, NKX2-1/TTF-1	Chromatin/Epigenetic, PI3K/AKT/mTOR
Oncogene	Oncogene	AKT1, BRAF, CTNNB1, EGFR, ERBB2/HER2, FGFR2/FGFR3, HRAS, KRAS, MET, NRAS, PIK3CA, RIT1, YAP1	Adhesion/EMT, Hippo/YAP, Immune modulation, PI3K/AKT/mTOR, RTK/RAS/MAPK, WNT/β-catenin
	activation	NFE2L2/NRF2	Immune modulation, Redox/NRF2
	amplification	CCNE1, MDM2	Cell cycle/RB, DDR
	contextual	PPFIBP1, RNF115	Adhesion/EMT
	fusion	ALK, NTRK1/2/3, RET, ROS1	PI3K/AKT/mTOR, RTK/RAS/MAPK
	neomorphic	IDH1	Metabolism/Cell stress
	promoter/amp	TERT	Telomere
	rare fusion/mutation	ERBB4	RTK/RAS/MAPK
	resistance	MAP2K1/MEK1	RTK/RAS/MAPK
	wt overexpress/rare mutation	IDH2	Metabolism/Cell stress, Redox/NRF2
	ligand (fusion)	NRG1	PI3K/AKT/mTOR, RTK/RAS/MAPK
RNA component		TERC	Telomere
RTK (contextual)		DDR2	Adhesion/EMT
Tumor suppressor	Tumor suppressor	APC, ARID1A, ATM, CDH1, CDKN2A, CHEK2, KEAP1, KMT2C, KMT2D, MGA, PIK3R1, PTEN, PTPRD, PTPRT, RB1, RBM10, SETD2, SMARCA4/BRG1, STK11/LKB1, TP53	Adhesion/EMT, Cell cycle/RB, Chromatin/Epigenetic, DDR, Immune modulation, Metabolism/Cell stress, PI3K/AKT/mTOR, Redox/NRF2, Splicing, WNT/β-catenin
	modifier	U2AF1	Splicing

## Data Availability

Not applicable.
